# Editorial: Thermophilic glycoside hydrolase from hot-spring microorganisms and its mechanism of high-temperature adaptation

**DOI:** 10.3389/fmicb.2024.1448900

**Published:** 2024-06-26

**Authors:** Yi-Rui Yin, En-Min Zhou, Hong Ming, Wael Nabil Hozzein, Iftikhar Ahmed, Hongchen Jiang, Wen-Jun Li

**Affiliations:** ^1^College of Agriculture and Biological Science, Dali University, Dali, Yunnan, China; ^2^International Joint Research Center for Karstology, Yunnan University, Kunming, Yunnan, China; ^3^College of Life Sciences and Technology, Xinxiang Medical University, Xinxiang, China; ^4^Botany and Microbiology Department, Faculty of Science, Beni-Suef University, Beni-Suef, Egypt; ^5^National Culture Collection of Pakistan, Land Resources Research Institute, National Agricultural Research Centre, Islamabad, Pakistan; ^6^State Key Laboratory of Biogeology and Environmental Geology, China University of Geosciences, Wuhan, Hubei, China; ^7^State Key Laboratory of Biocontrol, Guangdong Provincial Key Laboratory of Plant Resources and Southern Marine Science and Engineering Guangdong Laboratory (Zhuhai), School of Life Sciences, Sun Yat-Sen University, Guangzhou, China

**Keywords:** hot-spring, thermophilic glycoside hydrolases, bioinformatics analysis, metagenome, molecular dynamics simulations, high-temperature adaptation

In recent years, with the increasing global demand for sustainable development and bioenergy, the study of biocatalysts has become a hot topic in the field of biotechnology (Bertacchi et al., 2021). Glycoside hydrolases (GHs, EC 3.2.1) are enzymes that play a crucial role in the hydrolysis and synthesis of carbohydrate compounds (Lombard et al., 2014). They hydrolyze glycosidic bonds in monosaccharides, oligosaccharides, polysaccharides, saponins, and glycoproteins through endo- or exo-acting mechanisms and are widely used. In particular, thermophilic GHs from hot springs have emerged as ideal candidates for industrial and biotechnological applications due to their excellent stability and catalytic activity at high temperatures (Gomes et al., 2016). This Research Topic synthesizes recent research focusing on thermophilic GHs derived from hot spring microorganisms and their high-temperature adaptation mechanisms and discusses their potential value in biocatalysis and industrial applications.

As typical high-temperature habitats, hot springs harbor a rich and unique diversity of thermophilic microorganisms and thermophilic GHs, which hold great potential for industrial applications (Yang et al., 2017). However, due to the limitations of pure culture techniques in the laboratory, most microorganisms cannot be cultured, restricting our ability to explore thermophilic GHs (Uchiyama and Miyazaki, 2009; Hedlund et al., 2015). With the development of sequencing technology, we can now utilize bioinformatics analysis to detect a large number of potential GH genes directly from nucleic acid sequences based on genomics, transcriptomics and metagenomics (Sokal et al., 2022). Its function was then verified by heterologous expression and enzymatic properties. Combined with molecular dynamics simulations and experimental validation, in-depth exploration of the high-temperature adaptation mechanisms of thermophilic GHs is of significant importance for the discovery of new functional genes and the study of high-temperature adaptation mechanisms.

Currently, various thermophilic GHs from hot springs, including cellulases, β-glucosidases, xylanases, amylases, and chitinases, have been reported. In this Research Topic, Covington et al. used whole-genome analysis to detect a novel β-1,3-endoglucanase (Fsa16295Glu) of the GH50 subfamily from the aerobic hyperthermophile Fervidibacter sacchari, and Huang et al. identified a thermophilic GH1 β-glucosidase (LQBG8) from the total DNA of hot spring soil samples using metagenomics technology. Both exhibit thermophilic and thermostable properties as well as high resistance to inhibitors. These findings enrich our understanding of thermophilic GHs in hot springs and provide clues for the exploration of new biocatalysts.

Li et al. examined two xylanases, XynDRTY1 and XynM1, in hot springs through molecular dynamics simulations and evolutionary analysis and revealed the key regions involved in the high-temperature adaptation of xylanases, providing a theoretical basis for further enhancing the thermostability of xylanases. In Huang et al., molecular dynamics simulations of the β-glucosidase LQBG8 revealed the molecular basis of its thermophilic properties and attributed its stability to a reduction in conformational changes and an increase in structural rigidity. Structural modification studies on xylanase from *Myceliophthora thermophila* by Yang et al. demonstrated that truncation of its N- and C-termini could significantly improve the thermostability and catalytic activity of the enzyme. These results not only reveal the high-temperature adaptation mechanisms of GHs but also provide new strategies for future enzyme engineering and modification.

Future research should explore and discover more GHs from hot springs, especially those with unique functional and structural characteristics. In addition, by integrating multiomics analysis, protein engineering and computational biology techniques, the molecular mechanisms of GHs can be further elucidated, and more efficient and stable biocatalysts can be developed to meet the needs of industry and environmental protection.

In summary, research on thermophilic GHs from hot springs and their high-temperature adaptation mechanisms not only opens new avenues for biotechnology and industrial applications but also provides profound insights into the adaptation mechanisms of organisms in extreme environments. As research progresses, these enzymes are expected to play an increasingly important role in promoting sustainable development and bioenergy utilization.

## Author contributions

Y-RY: Funding acquisition, Writing – original draft, Writing – review & editing. E-MZ: Writing – review & editing. HM: Writing – review & editing. WH: Writing – review & editing. IA: Writing – review & editing. HJ: Writing – original draft, Writing – review & editing. W-JL: Funding acquisition, Writing – original draft, Writing – review & editing.
